# Iris-Claw Intraocular Lens: Anterior Chamber or Retropupillary Implantation? A Systematic Review and Meta-Analysis

**DOI:** 10.3390/medicina57080785

**Published:** 2021-07-30

**Authors:** I-Chia Liang, Yun-Hsiang Chang, Adrián Hernández Martínez, Chi-Feng Hung

**Affiliations:** 1 National Defense Medical Center, Department of Ophthalmology, Tri-Service General Hospital, Taipei 11490, Taiwan; ysonyaliang@gmail.com (I.-C.L.); yun.siang@me.com (Y.-H.C.); 2 Program in Nutrition and Food Science, Fu Jen University, New Taipei City 24205, Taiwan; 3 Department of Retina, Oftalvist Sevilla, 41007 Sevilla, Spain; oft.ahm@gmail.com; 4 School of Medicine, Fu Jen Catholic University, New Taipei City 24205, Taiwan; 5 Program in Pharmaceutical Biotechnology, Fu Jen Catholic University, New Taipei City 24205, Taiwan

**Keywords:** iris-claw, intraocular lens, anterior chamber, retropupillary

## Abstract

*Background and Objectives*: Iris-claw intraocular lens (ICIOL) could be implanted in the anterior chamber (AC) or retropupillary (RP) in eyes lacking capsular and/or zonular support. Several studies have focused on comparing the efficacy and complications of these two techniques and we designed this research to review the published literatures. *Materials and Methods*: Peer-reviewed studies were collected through network databases (PubMed, Scopus, Cochrane Central Register of Controlled Trials, and ClinicalTrials.gov) and analyzed. The primary outcome was the standardized mean differences (SMDs) of pre- and post-operative corrected distant visual acuity (CDVA). The secondary outcome was the SMDs of pre- and post-operative intraocular pressure (IOP), endothelial cell counts (ECC), and the odds ratios (ORs) of post-operative IOP elevation and cystoid macular edema (CME). Comprehensive Meta-Analysis software was utilized to conduct statistical analysis. *Results*: Six studies (one randomized controlled trial and five retrospective case series) were relevant and included a total of 516 eyes (255 and 261 eyes in the AC ICIOL and RP ICIOL groups, respectively). The quantitative analysis showed no significant differences in CDVA (SMD: 0.164, 95% confidence interval (CI): −0.171 to 0.500), ECC (SMD: −0.011, 95% CI: −0.195 to 0.173), and IOP elevation events (OR: 0.797, 95% CI: 0.459 to 1.383). Lesser IOP reduction (SMD: 0.257, 95%CI: 0.023 to 0.490) and a relative increase in the incidence of CME (OR:2.315, 95% CI: 0.950 to 5.637) were observed in the AC ICIOL group compared with RP ICIOL group. *Conclusions*: Our meta-analysis indicated that AC and RP ICIOL seem to have equivalent visual outcomes. RP ICIOL may perform slightly better with more IOP reduction and lesser CME. More randomized controlled trials, which have higher patient participation and more outcomes are needed to confirm our conclusions.

## 1. Introduction

Loss of the capsule and/or zonules can result for various reasons, including complicated cataract surgery, trauma, and ocular pathologies, such as pseudoexfoliation syndrome, Marfan syndrome, and lens coloboma. The correction of aphakia with inadequate support for the placement of a standard posterior chamber intraocular lens (PCIOL) in the capsular bag or in the ciliary sulcus is challenging for surgeons. Surgeons should weigh the pros and cons and make decisions after comprehensively considering their surgical techniques, available operating resources, and each patient’s condition.

Since the 1980s, 4-point fixation flexible open-loop angle-supported anterior chamber intraocular lens (ACIOL) have been used for the correction of aphakia in eyes without capsular support, and other alternative methods have been developed, including iris-fixation with iris-sutured intraocular lens and iris-claw intraocular lens (ICIOL) and scleral-fixation by suturing of PCIOL or intrascleral haptic fixation of PCIOL [1,2]. A recent report by the American Academy of Ophthalmology, published in 2020, concluded that the published evidence showed no superiority of any single surgical technique in the correction of aphakia without capsular support when comparing flexible open-loop ACIOLs, versus iris-claw aphakic lenses or lenses sutured to sclera. However, the researchers did not include in the assessment corneal edema or endothelial cell loss, because those data were not consistently reported in the studies [2].

The use of ACIOL has decreased during past decades because of its restrictions in younger patients and patients with glaucoma or uveitis for a relatively higher rate of corneal edema, secondary glaucoma, and uveitis [3,4]. Scleral-fixation requires superb and complicated surgical techniques and longer surgical times and is associated with more intraoperative and post-operative complications [5,6].

Suturing the intraocular lens (IOL) to the sclera or to the iris risks suture breakage, resulting in IOL tilt or dislocation of the IOL into the vitreous [7,8].

Worst et al. had published a technique using an iris-clip IOL, which required sutures to be fixed to the iris, in 1972 [9]. Artisan aphakic IOL (Ophtec BV), the first ICIOL without need of suture for correction of aphakia, was developed in 1978, and the phakic version for correction of myopia was introduced more than ten years later in 1989 [10,11,12]. Amar in 1980 published his technique using a modified iris-claw model to be placed in the retropupillary [13], and later Rijneveld et al. described their retropupillary fixation of ICIOL after cataract extraction during penetrating keratoplasty, in 1994 [14].These techniques with the IOL placed behind the iris, seemed to have the advantage of diminishing the potential risk of negatively affecting the corneal endothelium [15,16]. ICIOL implantation either in the anterior chamber or by retropupillary fixation became an effective and safe choice with relatively simple placement and better clinical outcomes compared with scleral-fixation intraocular lens or ACIOL [6,17,18,19]. Many studies focused on comparing the efficacy and complications of anterior chamber ICIOL (AC ICIOL) and retropupillary implantation ICIOL (RP ICIOL).

The purpose of this analysis was to review the published literature on AC ICIOL and RP ICIOL implantation in the absence of capsular/zonular support and to evaluate the outcome of corrected distant visual acuity (CDVA) and postoperative complications.

## 2. Materials and Methods

Studies comparing the outcomes of AC ICIOL and RP ICIOL implantation for aphakia with inadequate zonular and/or capsular support were systematically reviewed.

PubMed, Scopus, the Cochrane Central Register of Controlled Trials, and ClinicalTrials.gov were searched for studies on the use of anterior or retropupillary iris-claw IOL implantation for aphakia or insufficient posterior capsular support from the earliest record to December 2020. The bibliographies of included trials and related review articles were manually reviewed for relevant references. The terms of the search strategy were ‘‘iris-fix’’ OR ‘‘iris-claw’’ OR ‘‘iris-clip’’ OR ‘‘iris suture’’ OR ‘‘artisan’’ OR ‘‘verisyse’’ combined with ‘‘anterior’’ AND ‘‘retropupillary.’’ There was no language restriction. Regarding the types of included studies, randomized controlled trials (RCTs), comparative experimental trials, and retrospective case series (RCSs) were enrolled, whereas single-arm follow-up studies and case reports were excluded. All retrieved studies were required to comprise at least two arms and include anterior and retropupillary iris-claw IOL implantation. When more information was necessary, we contacted the authors by email.

All the retrieved articles and extracted data were examined independently by two specialists using a predetermined form that included the first author, publication year, study country, study design, sample size, number and type of treatment arms, participant characteristics and outcomes, and details of the ICIOL implantation procedures. The Jadad scoring for randomized studies and the Newcastle–Ottawa Quality Assessment Scale for nonrandomized studies were used to evaluate the methodological quality of the enrolled studies by two specialists independently. Between-reviewer discrepancies were solved through discussions under supervision of the corresponding author.

AC ICIOL and RP ICIOL implantations were compared in terms of primary and secondary outcomes. The primary outcome was the log MAR CDVA. Secondary outcomes were changes in intraocular pressure (IOP), the risk of an IOP elevation event, changes in endothelial cell counts (ECCs), and the risk of cystoid macular edema (CME).

Data were meta-analyzed using Comprehensive Meta-Analysis software, version 3 (Biostat Inc., Englewood, NJ, USA).

The primary outcomes were the standardized mean differences (SMDs) of pre- and post-operative CDVA between the AC ICIOL and RP ICIOL groups, and the secondary outcomes were the SMDs of pre- and post-operative IOPs and ECCs. The comparison was standardized by post-score standard deviation (SD). A negative SMD value indicated AC ICIOL to be a favorable treatment option, while a positive SMD favored RP ICIOL. Furthermore, the odds ratios (ORs) of post-operative IOP elevation events and CME in the AC ICIOL and RP ICIOL groups also represented secondary outcomes. We conducted all statistics with 95% confidence intervals (CIs).

Between-trial heterogeneity was determined using *I*^2^ tests; values >50% were regarded as considerably heterogeneous. A fixed-effects model would be chosen for those with an *I*^2^ < 50%, while a random-effects model would be used when *I*^2^ > 50%. Funnel plots and Egger’s test were used to examine potential publication bias. Both fixed- and random-effects models would be introduced if potential publication bias was noted. Statistical significance was defined as *p* < 0.05.

## 3. Results

### 3.1. Study Search and Characteristics of Included Patients

**Figure 1:**
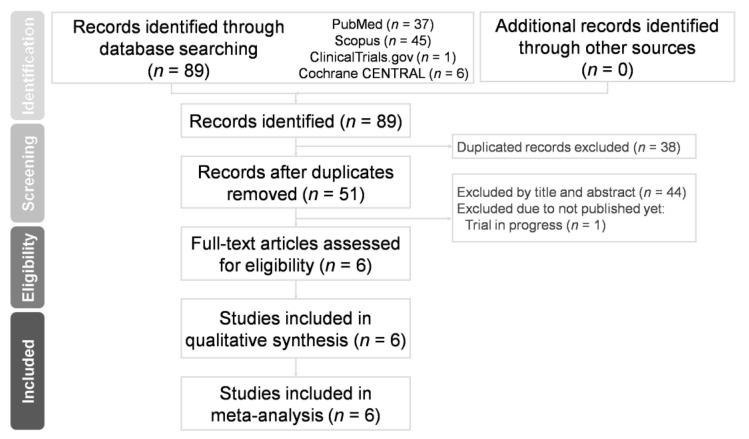
Preferred reporting items for systematic reviews and meta-analyses (PRISMA) flow diagram for the search and identification of included studies.

Fifty-one non-duplicate citations were retrieved for a review of their titles and abstracts, and 6 articles were included for meticulous evaluation after eliminating references violating the inclusion criteria (Figure 1). The meta-analysis included one RCT and five RCSs. In terms of the patient population, the RCT investigated patients with anterior chamber depths (ACDs) >3.2 mm and randomized to receive AC or RP ICIOL due to aphakia and a lack of capsular support, which resulted from trauma, complicated cataract surgery, and lens or IOL subluxation, while patients with glaucoma, iris defect, uveitis, and any pathology of the retina were excluded [20]. One RCS targeted patients who received secondary IOL implantation because of aphakia with a lack of posterior capsular support after cataract surgery, and patients who had a history of uveitis, proliferative diabetic retinopathy, and age-related macular degeneration and those who underwent any surgery except previous cataract surgery were excluded [21]. One RCS focused on cases with insufficient capsular support due to subluxated cataract, dislocated nucleus, subluxated or dislocated IOL, opacified IOL, or aphakia, and only patients with severe media opacity precluding examination of the ocular structures were excluded, while patients with pre-existing corneal, macular, or retinal pathologies and open or closed globe trauma were included [22]. One RCS included patients with aphakia secondary to complicated phacoemulsification, IOL dislocation, ocular trauma, crystalline lens subluxation in Marfan syndrome, and IOL opacification [23]. One RCS focused on participants undergoing ICIOL implantation due to trauma, subluxation of preexisting IOL, and lens subluxation related to pseudoexfoliation syndrome, Marfan syndrome, lens coloboma, or other pathologies with an exclusion of those with a history of ocular inflammation in the previous 6 months, uncontrolled IOP, severe corneal opacity, and poor visual prognoses [24]. One RCS included patients who underwent ICIOL implantation because of aphakia due to trauma, complicated cataract surgery and lens or IOL luxation, and excluded those with glaucoma, uveitis, retinopathies, and any ocular co-morbidity that was judged to interfere with the improvement in visual acuity; only 1 was randomly selected for inclusion if both eyes of the same patient had undergone ICIOL implantation [25]. Patient characteristics, study methodology, and quality assessment of the included trials are listed in Table 1, while Table 2 summarizes the surgical techniques in each study. Table 3 summarizes the results of all the analyses.

The final quantitative analysis included 516 participants.

### 3.2. Quality Assessment and Risk of Bias

**Figure 2:**
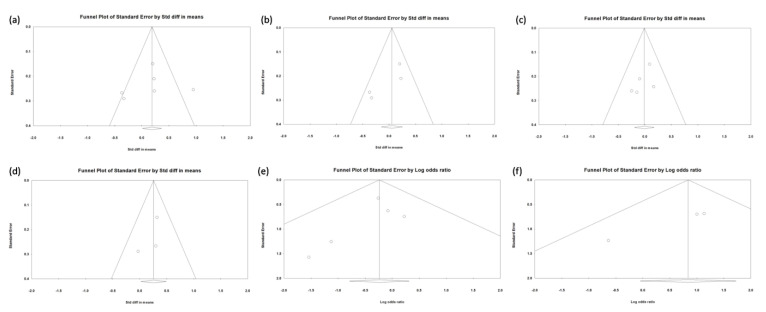
(**a**) Funnel plot of CDVA changes (6 studies). (**b**) Funnel plot of CDVA changes (4 studies). (**c**) Funnel plot of ECC changes. (**d**) Funnel plot of IOP changes. (**e**) Funnel plot of IOP elevation events rates. (**f**) Funnel plot of CME rates.

The quality score of the 6 studies according to the Newcastle–Ottawa scale or the Jadad scoring is displayed in Table 1 and indicated a low-to-moderate risk of bias. The Egger’s test revealed no significant publication bias regarding the overall SMD and ORs, except for the OR of the CME event. The funnel plots are shown in Figure 2.

### 3.3. CDVA and ECC Outcomes between AC and RP ICIOL

**Figure 3:**
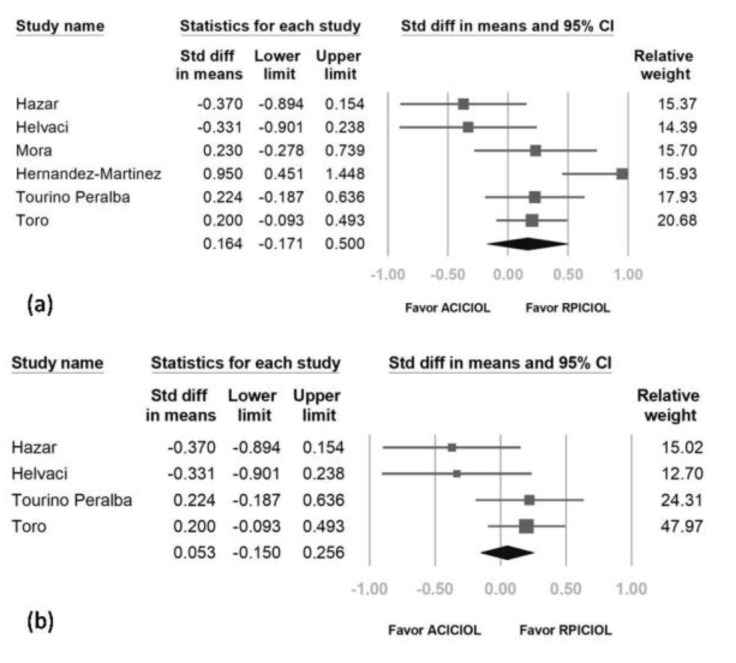
Forest plot of corrected distant visual acuity of all 6 studies (**a**) and after removing 2studies with larger heterogeneity and potential bias (**b**).

The overall SMD of AC ICIOL versus RP ICIOL regarding pre- and post-operative CDVA was 0.164 (95% CI: −0.171 to 0.500) (Figure 3a). Regarding SMD heterogeneity, the *I*^2^ was 69.855%. The preoperative CDVA was low and resulted in larger differences between pre- and post-operative CVDA in one study, in which many patients underwent surgery for phacoemulsification complications at the same time as they received an ICIOL implant [23]. Another study includes patients with corneal and retinal pathologies, which may alter the CDVA [22]. After removing these two studies, the overall SMD of the pre- and post-operative CDVAs of the other 4studies was 0.053 (95% CI: −0.150 to 0.256) (Figure 3b) with an *I*^2^ of 49.051.

**Figure 4:**
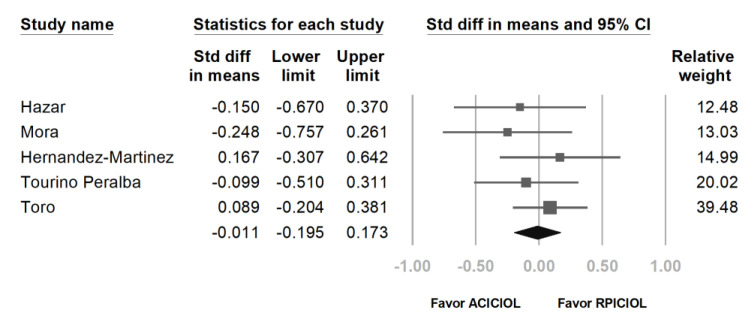
Forest plot of endothelial cell counts.

The overall SMD of the pre- and post-operative ECC was −0.011 (95% CI: −0.195 to 0.173) (Figure 4) with an *I*^2^ of less than 0.001%.

### 3.4. IOP Outcome and Risk of IOP Elevation between AC and RP ICIOL

**Figure 5:**
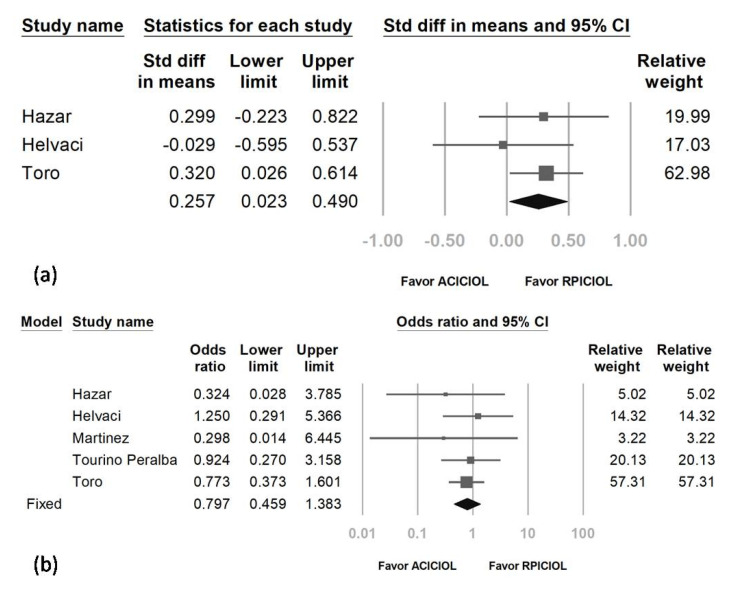
Forest plot of intraocular pressure (IOP) (**a**) and IOP elevation event rate (**b**).

The result relative to pre- and post-IOP showed that IOP was reduced less by AC ICIOL than by RP ICIOL. The overall SMD was 0.257 (95% CI: 0.023 to 0.490) (Figure 5a). Regarding SMD heterogeneity, *I*^2^ was less than 0.001%.

The pooled OR of IOP elevation events (either with IOP elevation requiring medication [23], IOP elevation noted within the first post-operative week [20] or the first post-operative month [24], or IOP more than 21 or 22 for more than 1 week [21,25]) in the AC ICIOL arm compared with the RP ICIOL arm was 0.797 (95% CI: 0.459–1.383 with the fixed- or random-effect model) (Figure 5b). Regarding the heterogeneity, the *I*^2^ was less than 0.001%.

### 3.5. Risk of CME between AC and RP ICIOL

**Figure 6:**
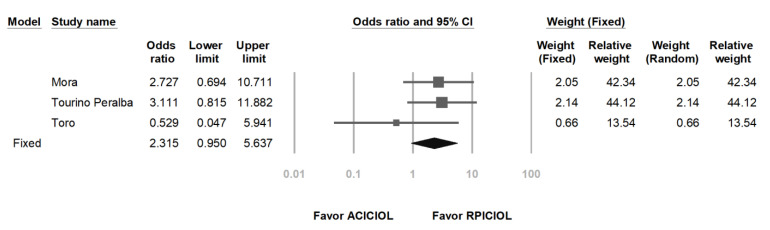
Forest plot of central macular edema rate.

The pooled OR of CME in the AC ICIOL arm compared with the RP ICIOL arm was 2.315 (95% CI: 0.950–5.637 with the fixed- or random-effects model), indicating a probably increased incidence of CME following AC ICIOL (Figure 6). Regarding the heterogeneity of the OR, the *I*^2^ was less than 0.001%.

## 4. Discussion

Both techniques have advantages and disadvantages regarding their difficulty and associated complications. Although previous studies have focused on the surgical procedures and outcomes of AC ICIOL and RP ICIOL implantations, the results and conclusions are controversial.

In terms of our primary outcome, there was no significant difference in visual acuity changes between the AC ICIOL and RP ICIOL groups in the 6 enrolled studies. In addition, in all the studies, good post-operative visual improvement was achieved in both groups; therefore, both alternatives perform equally well in ameliorating visual acuity, and it is reasonable to infer this result with the good centration and stability of both AC and RP ICIOL.

A major concern about using AC ICIOL is the long-term effect on ECC due to its proximity to the corneal endothelium. Progressive ECC loss is reported in phakic AC ICIOL. Galviset al. reported an ECC loss of more than 25% of the pre-operative ECC in one-fifth of eyes [11]. The ECC decrease remained progressive throughout the 3-to-5-year follow-up period [26,27]. Similar annual ECC loss was also reported for AC ICIOL implantation for aphakic eyes in both adults and children [28,29]. The influence of RP ICIOL on ECC does not seem significant, inasmuch as Forlini et al. reported no significant change in ECC after RP ICIOL implantation after a mean follow-up period of 5.3 years [16]. Data about annual ECC changes after RP ICIOL implantation are few. Choi et al. reported that ECC was significantly reduced compared with the pre-operative amount one month after RP ICIOL implantation but stabilized thereafter up to 2 years of follow-up [30]. However, Anbari and Lake reported gradual ECC loss in a 2-year follow-up [31]. In our meta-analysis, the ECC decreased in both the AC and RP ICIOL groups between pre-operative and last visits in 5enrolled studies, but there was no significant difference in overall ECC changes noted between the two groups. However, only 2 studies enrolled had follow-up periods longer than 2 years, which were 33 ± 21.8months [23] and 60 months [25]. The follow-up period of the other 3studies was between 6 and 12 months. More analyses focused on the ECC before, just after the operation, and annually afterwards are necessary to understand the exact influence of AC or RP ICIOL implantation on ECC.

Regarding IOP, there was no significant difference in the risk of IOP elevation events between the two groups, even if RP ICIOL seemed to result in lower IOPs after surgery compared with pre-operative IOPs. This discrepancy could be explained by the slight elevation of IOPs in the AC ICIOL group in some studies. As a result, RP ICIOL may be better for eyes with ocular hypertension or borderline IOP before surgery. Performing a peripheral iridotomy has been recommended when the ICIOL is placed on the anterior surface of the iris to prevent papillary block and subsequent IOP elevation problems; however, that procedure may be unnecessary with the lens positioned behind the iris [22,25,32]. Peripheral iridotomies could also widen the iridocorneal angle [33,34,35]. Peripheral iridotomies were performed for ACICIOL but not for RPICIOL in most cases included in the analysis and the results showed equal risk of IOP elevation event between the two groups.

CME has been reported to occur after ICIOL implantation. The exact cause is not clear but may be due to subclinical chronic low-grade irritation of the iris [36], or even just a result of the primary cause of aphakia or the vitreoretinal surgery itself [37]. The risk of CME seemed to be higher in the AC ICIOL group, although it was not significant. The difference may be caused by the different grade of inflammation, or pigment dispersion between the anterior and posterior iris surfaces. Another possibility may be the hypothesis of post-ICIOL CME proposed by Massa et al. [36], which is that insufficient iris tissue may be captured in the claw, resulting in the movement of the IOL against the iris; RP ICIOL might be able to enclave more iris tissue than AC ICIOL.

There are several limitations to our study.

Due to the restriction of the ACD for the implantation of AC ICIOL, most studies included in this meta-analysis were not randomized, except for that of Helvaci et al. [20], which included eyes with ACD > 3.2mm only to avoid the contraindication of AC ICIOL.

In all included studies, there was no detailed information reported about the post-operative medication, such as systemic or topical anti-inflammatory agents (non-steroidal anti-inflammatory drugs, corticosteroids, etc.) and the treatment duration. However, these post-operative treatments could make an impact on IOP and even the risk and severity of CME.

There are other clinically significant complications of ICIOL implantation techniques, including wound leakage, hyphema, severe inflammation, and IOL malpositioning (including pupillary IOL optic capture, decentration, tilt, or dislocation) shortly after the operation; long-term complications include glaucoma, chronic uveitis, and even IOL subluxation or dislocation. Besides, both techniques fixed IOL on the iris. ICIOL might have a higher rate of various complications that are related to the iris and pupils compared with other IOL-fixation methods for insufficient capsular/zonular support, including iris atrophy, pigment deposition, pigment dispersion, and pupillary distortion. These complications, as well as the centration of ICIOL and ICIOL positioning just after the operation, were not discussed in this review.

## 5. Conclusions

The present meta-analysis revealed that, with rigorous pre-operative assessment, AC ICIOL and RP ICIOL had equal effects on visual recovery. However, RP ICIOL may perform better with greater IOP reduction and reduced CME. Subsequent RCT swith more participants and more outcomes are needed to confirm our conclusions. More comprehensive and detailed complication analyses with longer follow-ups are necessary to understand more about the difference between these two procedures.

## Figures and Tables

**Table 1:** Characteristics of Studies Included in the Meta-Analysis.

Author, Year	Country	Study Design	Enrolled Sample Number, Eyes (Male/Female)	Age Mean ± SD, Years	Outcome Measurement	Follow-Up Time, Months	Quality Assessment
Hazar et al., 2013	Turkey	RCS	AC ICIOL: 35 (24/11) RP ICIOL: 24 (14/10)	AC ICIOL: 52 ± 18 RP ICIOL: 61 ± 16	CDVA, IOP, slit-lamp exam, fundus exam, ECC, FA (if needed)	AC ICIOL: 12.0 ± 7.7 CIOL: 10.1 ± 7.3 (mean ± SD)	4 **
Helvaci et al., 2016	Turkey	RCT	AC ICIOL: 20 (11/9) RP ICIOL: 30 (13/7)	AC ICIOL: 68.5 ± 6.8 RP ICIOL: 69.9 ± 8.2	CDVA, IOP, slit-lamp exam, fundus exam	6	1 *
Mora et al., 2018	Italy	RCS	AC ICIOL: 28 (20/8) RP ICIOL: 32 (21/11)	AC ICIOL: 72.7 ± 13.5 RP ICIOL:73.8 ± 13.4	CDVA, IOP, slit-lamp exam, fundus exam, ECC, CMT on OCT	12	4 **
Hernández-Martínez et al., 2018	Spain	RCS	AC ICIOL: 28 (14/14) RP ICIOL: 44 (23/21)	AC ICIOL: 74.0 ± 14.0 RP ICIOL: 70.4 ± 12.8	UDVA, CDVA, SIA, ECC	33 ± 21.8 (mean ± SD)	2 **
Tourino Peralba et al., 2018	Spain	RCS	AC ICIOL: 57 (32/25) RP ICIOL: 38 (20/18)	AC ICIOL: 66 ± 15.6 RP ICIOL: 72.5 ± 11.1	CDVA, IOP, slit-lamp exam, fundus exam, corneal astigmatism, ECC, CMT on OCT	12	3 **
Toro et al., 2019	Italy	RCS	AC ICIOL: 87 (49/38) RP ICIOL: 93 (53/40)	AC ICIOL: 70.6 ± 5.5 RP ICIOL: 69.5 ± 6.3	CDVA, IOP, slit-lamp exam, fundus exam, CCT, ECC, CMT on OCT	60	4 **

AC ICIOL: anterior chamber iris-claw intraocular lens, CCT: central corneal thickness, CDVA: corrected distance visual acuity, CMT: central macular thickness, ECC: endothelial cell count, FA: fluorescein angiography, IOP: intraocular pressure, OCT: optical coherence tomography, PPV: pars plana vitrectomy, RCS: retrospective case series, RCT: randomized controlled trial, RP ICIOL: retropupillaryiris-claw intraocular lens, SD: standard deviation, UDVA: uncorrected distance visual acuity. * indicated that the study was evaluated by Jadad’s scale. ** indicated that the study was assessed by the Newcastle–Ottawa Scale.

**Table 2:** Details of surgical techniques.

Author, Year	ICIOL	Main Wound Location	Side Ports Location	Vitrectomy	Instrument for Iris Fixation	Peripheral Iridotomy	Wound Closure
Hazar et al., 2013	Artisan or Verisyse	Superior 6.0mm clear corneal incision	AC ICIOL: 2 and 10 o’clock RP ICIOL: 3 o’clock for left eye/9 o’clock for right eye	Anterior vitrectomy if vitreous in AC	AC ICIOL: enclavation needle RP ICIOL: Sinskey hook or iris spatula	AC ICIOL: 12 o’clock RP ICIOL: not performed	Interrupted 10-0 nylon
Helvaci et al., 2016	Artisan	Superior 5.5 mm limbal corneal incision	2 and 10 o’clock	Anterior vitrectomy or PPV for cases with IOL luxation, lens luxation, luxated nigra cataract	Enclavation needle	AC ICIOL: superior RP ICIOL: not performed	Interrupted sutures
Mora et al., 2018	Artisan	Superior 5.5 mm limbal corneal incision	3 and 9 o’clock	Anterior vitrectomy as required or PPV if indicated	Enclavation microspatula	AC ICIOL: superior RP ICIOL: not performed	Noncontinuous 10-0 nylon
Hernández-Martínez et al., 2018	Artisan	Superior 5.5 mm corneal incision or scleral tunnel 2.0 mm from the limbus	2 and 10 o’clock	PPV or anterior vitrectomy through corneal access in eyes that did not require a PPV	AC ICIOL: 16 cases- claw needle/12 cases- Vacufix system (Ophtec BV) RP ICIOL: claw needle	AC ICIOL: 12 o’clock RP ICIOL: not performed	AC ICIOL: 5 cases- scleral incision(Interrupted 10-0 nylon)/23 cases- corneal incision (Continued 10-0 nylon) RP ICIOL: 31 cases- scleral incision (Interrupted 10-0 nylon)/13 cases- corneal incision (Continued 10-0 nylon)
Tourino Peralba et al., 2018	Artisan	Superior clear corneal incision	AC ICIOL: 2 and 10 o’clock RP ICIOL: 3 o’clock for left eye/9 o’clock for right eye	Extensive anterior vitrectomy if no previous peripheral iridectomy performed previously	AC ICIOL: enclavation needle RP ICIOL: reverse Sinskey hook or 27-gauge needle bent 45 degrees	Most cases: peripheral iridectomy performed during previous procedures Remaining cases: not performed	Interrupted 10-0 nylon
Toro et al., 2019	Artisan	Superior 5.5 mm limbal corneal incision	3 and 9 o’clock	Anterior vitrectomy as required	Enclavation microspatula	AC ICIOL: superior RP ICIOL: not performed	Interrupted 10-0 nylons

AC: anterior chamber, IC: iris-claw, IOL: intraocular lens, PPV: pars plana vitrectomy, RP: retropupillary, SD: standard deviation.

**Table 3:** Summary of the results of all the analyses.

Analysis	No. of Studies	Subjects (No.)	SMD/OR	95% CI	*p* Value for Differences	Heterogeneity		Egger’s Test
						*p* Value	*I* ^2^	*p* Value
CDVA	6	516	0.164	−0.171, 0.500	0.337	0.005	69.855	0.741
CDVA	4	384	0.053	−0.151, 0.256	0.610	5.888	49.051	0.136
ECC	5	466	−0.011	−0.195, 0.173	0.908	0.686	0.000	0.290
IOP	3	289	0.119	0.023, 0.490	0.031	0.553	0.000	0.488
IOP elevation event	5	444	0.788	0.454, 1.368	0.398	0.801	0.000	0.339
CME event	3	347	2.315	0.950, 5.637	0.065	0.433	0.000	0.044

CDVA: corrected distance visual acuity, CI: confidence interval, CME: central macular edema, ECC: endothelial cell count, IOP: intraocular pressure, No.: number, OR: odds ratio, SMD: standardized mean difference.

## Data Availability

The datasets generated and/or analyzed during the current study are available from the corresponding author on reasonable request.
